# Editorial: The welfare of working animals

**DOI:** 10.3389/fvets.2023.1339792

**Published:** 2024-01-08

**Authors:** Katarina Nenadović, Tomislav Mikuš

**Affiliations:** ^1^Faculty of Veterinary Medicine, University of Belgrade, Belgrade, Serbia; ^2^Faculty of Veterinary Medicine, University of Zagreb, Zagreb, Croatia

**Keywords:** working animals, welfare, working dogs, equids, stunning heavy cattle, camelids, donkeys

This Research Topic gathers different contributions highlighting the latest research on the welfare of working animals. Articles ranged from working dogs, camelids, equids to heavy cattle, so we can say that contributors have fulfilled our intention, which was to identify problems, spread knowledge and seek solutions to further improve the welfare of all working animals. However, two groups of animals were more significantly represented, the first one being working dogs, and the other working equids.

Working dogs are prevalent around the world and fulfill many roles, adding social, cultural and economic value to human lifestyles. Dogs work in herding, guarding, hunting, human assistance, and animal-assisted therapy. In the last decade, our understanding of working dog performance and animal welfare science has grown rapidly. Despite this, there remains a great deal of room for further research, development, and improvement in working dog welfare (1).

In the first article in the working dog's group by Farr et al. a revised version of Penn Vet Working Dog Center Sprint Test is presented. This study illustrates the validity of assessing performance by naive and experienced raters, demonstrates that dogs accelerate similarly to humans, and discusses the performance of some working dogs. These outcomes are expected to establish the aforementioned sprint test as a valid measure of canine fitness, facilitate its use in future research, and enhance the medical care and welfare of canine athletes.

Earnshaw et al. have presented the first ever research examining the health of working dogs used in conservation in Africa. Authors emphasize that the importance of the health and welfare of these highly valuable dogs has been overlooked in the published literature as they require a unique skill set to be successful in their work. A strong handler-dog attachment, proficient handler training, and the acknowledgment of the challenging environment were pivotal to maintaining dog health. Finally, we believe that this very specific study will give additional help to those working in conservation programmes giving them a more comprehensive insight into what is required to establish and maintain a conservation dog programme.

The final two articles on the working dog welfare are focused on issues regarding their coat. On the one side, Discepolo et al. presents the problems of overbathing the working dogs and the potential health and welfare problems which can arise from this practice. The results of their presented work show that repetitive bathing of canines with detergent resulted in significant impacts on the resident dermal microbiota on canine skin. The study by Perry et al. gives an additional insight into the issues regarding the health concerns associated with potential bacterial cross-contamination from working canines to humans. The authors have selected specific method of decontamination—wipe-down procedure, as it may be more readily utilized for canines working in healthcare setting to prevent the spread of bacteria, but the method can also be used easily in other scenarios, such as natural disasters. The study clearly states that both tested solutions (povidone-iodine and chlorhexidine gluconate) have similarly effective biocidal activity on the canine coat, both are easily available and approved for veterinary use, and easy to acquire and safe for utilization in canines.

Equids are still one of the most important resources for countless families around the world, who use them in a number of jobs, from cultivating fields to transporting goods (2). In this Research Topic, the welfare of working equids is represented by three articles, two research studies and a one systematic review. The cross-sectional study of donkey owners in Pakistan by Bukhari et al. provides evidence of on-the-ground working practices and factors associated with mounted load carrying, which is critical for developing evidence-based recommendations for loading, to improve the welfare of working donkeys. This study confirmed the necessity for future education of owners regarding the overloading as most donkeys reported in the study carried more than the recommended 50% limit of their bodyweight ratio.

The article by Cousquer et al. on the history and welfare of mules engages with systems thinking and presents their welfare as a complex interaction of several elements. Some of these elements are material, related to the animal itself and the tack, others are historical, geographical, socio-cultural, socio-economic and psychological. This article therefore deliberately sets out to highlight the emergent complexity of such relational systems and the need for our thinking to move away from linear cause-and-effect thinking and to embrace, instead, the systems approach with regard to One Health.

The review by Bukhari and Parkes focuses on the biomechanical, physiological, biochemical, and behavioral impacts of pulling load on equids and their welfare. Authors presents details all of the above-mentioned factors with special emphasis on the usage of simple indicators such as eye blink rate as one of the indicators of stress which could be used in the future assessments of overloading.

Kandeel et al. presented in their systematic review a bibliometric analysis of camel research. In their comprehensive article, the major contributors to camel research throughout the past century are discussed, along with the funding sources, academic institutions, scientific disciplines, and countries that contributed to the selected topic.

Finally, the last article by Gascho et al. focuses on the stunning of heavy cattle due to the problems that can occur at this sensitive moment due to their very thick frontal bones. In their research authors tested a new method and bullets for stunning heavy cattle. Based on the results of this study, the authors recommend two types of bullets for stunning heavy cattle with BigBovid - Hornady FTX and Hydra-Shok. These types of bullets have a high energy density and therefore a high penetration potential through the thick frontal bones of heavy cattle, secondly, excessive penetration is unlikely due to high fragmentation and thirdly at the relevant penetration depth, these two types of bullets caused adequate cavity volume and are therefore considered suitable for stunning heavy cattle.

## Author contributions

KN: Writing—review & editing. TM: Conceptualization, Writing—original draft, Writing—review & editing.
